# Between fiscal returns and social harm: reframing gambling regulation in Kazakhstan through international evidence

**DOI:** 10.3389/fsoc.2025.1718858

**Published:** 2026-01-08

**Authors:** Zhanna Khamzina, Yermek Buribayev

**Affiliations:** Department of Law, Zhetysu University named after I. Zhansugurov, TaldyKorgan, Kazakhstan

**Keywords:** comparative public policy, gambling regulation, harm-reduction measures, regulatory instruments, social costs of gambling

## Abstract

Background Since 2007, Kazakhstan has gradually modernized gambling regulation and in 2023–2024 enacted major reforms, including the creation of a specialized regulator, tighter advertising rules, expanded exclusion regimes, and stronger criminal enforcement. Objective: To assess how this evolving regulatory model aligns with international practice and to derive evidence-informed policy options, treating gambling law as a sociolegal regime for allocating gambling-related risks and fiscal rents. Methods: We use an exploratory sociolegal mixed-methods design that combines doctrinal and comparative legal analysis with a descriptive synthesis of 2019–2024 official statistics on market size, taxation, enforcement, and harm-reduction proxies. Results Kazakhstan’s legal gambling market expanded further in 2024, recorded criminal cases for illegal gambling declined, and coverage of debtor-based and voluntary self-exclusion instruments increased sharply, while a large offshore online segment persisted. Situating these trends within a comparative coding of the United Kingdom, Spain, and Italy, we show that Kazakhstan combines strict advertising controls, extensive exclusion tools, and advanced enforcement technologies with a comparatively heavy and complex tax mix and a regulator located within a line ministry. Conclusion A public-health-oriented configuration of instruments appears normatively most consistent with the dual goals of channeling demand into the legal market and mitigating gambling-related harm, but the available data remain descriptive and the post-reform observation window is short. We discuss incremental policy options regarding taxation, regulator autonomy, harm reduction, and cross-border enforcement.

## 1 Introduction

From a theoretical standpoint, we draw on sociolegal approaches to risk regulation that conceptualize law as a central medium for allocating social risks and rents (Beck, 1992; Ewald, 1991; Braithwaite, 2005). Within this framework, we develop an ideal-typical classification of gambling regulation distinguishing among liberal, restrictive, and prohibitive models (Buribayev and Khamzina, 2025a). Liberal regimes are characterized by broad market access for licensed operators, relatively moderate taxation, and a primary emphasis on competition and consumer choice, subject to standard responsible-gambling requirements. Restrictive regimes permit gambling within a clearly delineated legal perimeter but deploy tighter constraints on availability, product scope, marketing, and price. Prohibitive regimes, by contrast, either ban most commercial gambling outright or confine it to narrow, state-controlled niches. These models are ideal types rather than empirical descriptions: most jurisdictions combine elements of more than one model, and their regulatory trajectories can be understood as movement within this conceptual space. In sociolegal terms, the resulting configurations can be read as institutionalized compromises between the state, markets, and households over the legitimate distribution of gambling-related harm and fiscal revenues.

Applied to Kazakhstan, this typology allows us to interpret the country as an evolving mixed configuration with pronounced restrictive and partially prohibitive features. The geographically zoned land-based casino and slot-hall model, combined with the de jure prohibition of online casino-style gambling and other unlicensed remote formats, positions the country closer to the restrictive/prohibitive end of the spectrum, even as recent reforms have expanded the legal market and modernized regulatory instruments. At the same time, the 2023–2024 reforms—including the creation of a specialized regulator, the introduction of comprehensive exclusion regimes and strict advertising controls, and the use of centralized bet accounting and payment blocking—align Kazakhstan with European jurisdictions such as Spain and Italy, which similarly combine stringent advertising bans and extensive consumer-protection portfolios with relatively heavy effective tax burdens. By contrast, the United Kingdom represents a more liberal, yet institutionally highly developed, model built around gross gambling revenue (GGR)-based taxation and a broad online licensing perimeter. The typology thus provides a coherent theoretical lens for the subsequent comparative analysis of Kazakhstan, the United Kingdom, Spain, and Italy, and for assessing Kazakhstan’s position within the wider international landscape of gambling regulation.

Since the adoption of the Law “On the Gambling Business” (No. 219-III, January 12, 2007), Kazakhstan has followed a geographically selective legalization strategy. Casinos and slot halls may operate only in designated zones in the Shchuchinsk district (Akmola region) and along the Kapshagay Reservoir in the city of Konaev, while bookmakers and totalizators may operate nationwide subject to licensing. By the end of 2024, 28 licenses covered casinos, slot-machine halls, bookmakers, and totalizators, and the sector made a nontrivial contribution to employment and tax revenues; for example, the Kapshagay/Konaev gambling zone alone generated roughly 96 billion KZT in tax revenues and around 6,000 jobs in 2023 (Buribayev and Khamzina, 2025b). Under the same law, online casino-style gambling and other unlicensed remote formats remain prohibited and therefore fall outside the national licensing perimeter; throughout this article we use the term “illegal gambling” to denote such unlicensed activities, including offshore online operators serving Kazakhstani residents.

The institutional foundations of regulation have been strengthened in parallel with market growth. In 2024, a new regulatory authority—the Committee for the Regulation of Gambling and Lottery Activities under the Ministry of Culture and Sports—was established with broad oversight powers. In cooperation with other agencies, the Committee initiated more than 100 cases against illegal gambling operations and identified over 11,000 unlawful websites in 2024. Amendments to the Law on Gambling that entered into force in September 2024 further enhanced consumer protection and standardized rules for all forms of gambling. Notably, advertising restrictions were significantly tightened, banning most outdoor and mass-media advertising for bookmakers, and a national self-exclusion mechanism was introduced; by the end of 2024, the voluntary exclusion register covered more than 170,000 citizens. Together, these measures indicate an explicit policy shift toward a more public-health-oriented regulatory configuration.

Despite these regulatory and fiscal developments, the social costs associated with gambling remain substantial. Official estimates suggest that approximately 350,000 Kazakhstani citizens exhibit signs of problem gambling, with particularly rapid growth among young people: the proportion of youth who regularly participate in gambling rose from 0.1% in 2021 to 6.3% in 2024, and the average debt of a problem gambler exceeds USD 22,000 (Buribayev and Khamzina, 2025b). Excessive gambling is associated with over-indebtedness, relationship breakdown, and related health problems, and survey evidence indicates that more than one-third of young respondents attribute family dissolution at least partly to a partner’s gambling addiction. In the international literature, such outcomes are conceptualized as “gambling-related harm” and, where monetized, as “social costs”; in this article we use these terms in this standard sense and operationalize them through a limited set of proxy indicators discussed in Section 2.2.

Alongside the legal market, a sizeable shadow sector persists, primarily consisting of offshore online casinos and unlicensed betting websites, with expert estimates placing its turnover at roughly four to five times that of the legal market (Caravan.kz, 2023). This configuration implies significant revenue losses for the state and heightened risks for players, who are unprotected against fraud and lack access to formal dispute-resolution and harm-reduction mechanisms. More broadly, the continued prominence of the shadow sector, incomplete consumer protection, and gaps in industry data indicate that Kazakhstan’s regulatory framework remains in transition and that the allocation of gambling-related risks between the state, licensed operators, and households is still being renegotiated.

Internationally, analogous challenges have prompted the gradual development of public-health-oriented regulatory architectures that combine legalization within a circumscribed perimeter with portfolios of harm-reduction instruments, including rigorous licensing, age and identity verification, self-exclusion and exclusion of specific high-risk groups, advertising controls, and the deployment of digital monitoring technologies (Wardle et al., 2024; Ukhova et al., 2024a, 2024b; Wardle et al., 2024; World Health Organization, 2023). While causal evidence remains contested and even relatively advanced systems continue to struggle with novel online products and marketing channels, these approaches provide a relevant comparative benchmark for assessing Kazakhstan’s recent reforms and for deriving policy lessons. In what follows, we therefore situate Kazakhstan’s evolving regulatory mix within this wider international repertoire of instruments and configurations.

## 2 Methodology

### 2.1 Design and data

We use an integrative sociolegal and descriptive empirical design that combines doctrinal and comparative legal analysis with a quantitative synthesis of official statistics. The legal component examines Kazakhstan’s gambling legislation and policy documents in parallel with regulatory frameworks in three European jurisdictions—the United Kingdom, Spain, and Italy. The empirical component is an annual country-level panel for 2019–2024 that tracks market development, fiscal returns, enforcement activity, and harm-reduction coverage. For each year we assemble three indicator blocks: (i) legal gambling services volume and gambling-specific tax receipts; (ii) criminal cases under Article 307 of the Criminal Code on illegal gambling (including the revised offense for operating online casinos); and (iii) harm-reduction indicators—voluntary self-exclusions and statutory exclusions for debtors and public officials. Data are drawn from national statistical and fiscal authorities, the sectoral gambling regulator, and related official documents, and are aggregated to annual totals. We compute simple year-on-year (y/y) changes and pre−/post-reform contrasts centered on the 2023–2024 reforms. For the estimated size of the offshore online market (around four to five times the legal segment), we rely on expert assessments reported in national media and policy documents; these figures are treated as contextual and interpreted with caution given the absence of transparent methodologies. Overall, the analysis is explicitly descriptive and exploratory: the short post-reform window and data limitations preclude formal causal identification.

### 2.2 Operationalization of social harm and social costs

In line with the public-health literature, we understand “gambling-related social harm” as the bundle of health, financial, and social harms associated with gambling at individual, family, and community levels, and “social costs” as the monetized subset of these harms. Kazakhstan does not yet produce a comprehensive social-cost account for gambling, and clinical statistics substantially under record gambling disorder; a full cost-of-illness estimation is therefore not feasible. Instead, we treat a limited set of indicators as proxies. Market development and legal-market channelization are approximated by legal gambling services volume and gambling-specific tax receipts, complemented by enforcement outputs (criminal cases and blocked websites/payments), which indicate the extent to which gambling demand is captured by the regulated sector rather than illegal or offshore operators. Harm-reduction coverage is proxied by voluntary self-exclusions, debtor-based exclusions, and bans on public officials. These measures are interpreted primarily as institutional responses to gambling-related harm—their increase signals an expansion of protective coverage but does not by itself imply a reduction in underlying prevalence or severity. Throughout the paper we therefore triangulate trends in these proxies with the doctrinal and comparative analysis of regulatory instruments and refrain from strong causal claims about effects on total social costs.

### 2.3 Comparative coding and analytical strategy

To situate Kazakhstan within a wider regulatory landscape, we complement the national trends with a structured qualitative comparison of selected European jurisdictions. We select the United Kingdom, Spain, and Italy as large, data-rich European markets that (a) have implemented major reforms to online gambling in the past 10–15 years and (b) span a spectrum from moderate to very strict advertising restrictions. This makes them suitable comparators for Kazakhstan’s evolving regime, which combines tight advertising controls, extensive exclusion tools, and robust enforcement technologies. We consciously do not extend the comparative coding to other post-Soviet or regional jurisdictions at this stage, because systematic and comparable data on key regulatory instruments and harm indicators remain limited for many of them, and their current regulatory trajectories differ markedly from the mature, online-focused European markets that constitute our primary reference frame.

We code each jurisdiction on a 0–2 scale along five regulatory instruments that recur in the international literature and in Kazakhstan’s recent reforms:

- Tax model (0 = high fixed/complex burden, 1 = mixed regime, 2 = predominantly competitive GGR-based taxation).
- Advertising restrictiveness (0 = relatively lenient, 1 = moderate restrictions; 2 = strict or near-ban regime).
- Protection coverage (0 = minimal tools; 1 = intermediate coverage; 2 = broad, national self-exclusion and targeted bans).
- Enforcement technology (0 = basic enforcement; 1 = systematic website blocking; 2 = website and payment blocking and/or centralized bet accounting).
- Regulator autonomy (0 = fragmented or weak oversight; 1 = specialized unit within a ministry; 2 = independent agency with statutory consumer-protection and enforcement mandates).

Foreign entries are coded based on statutory texts, regulator reports, and secondary analyses cited in the article. Coding was performed by the authors; disagreements were resolved through discussion. The rubric is purely descriptive and is not used for formal causal inference. Rather, it provides a transparent framework to locate Kazakhstan’s regulatory mix relative to better-documented European regimes and to structure the subsequent discussion of which elements of foreign practice appear most transferable to the Kazakhstani context. Table 1 summarizes the coding.

**Table 1:** Instrument coding.

Jurisdiction (year)	Tax model	Advertising restrictiveness	Protection coverage	Enforcement technology	Regulator autonomy
Kazakhstan (2024)	0–1 (mixed; high effective burden including GGR + VAT + fixed rates)	2 (very strict)	2 (broad: debtor + public-official + voluntary self-exclusions register)	2 (systematic website and payment blocking; centralized bet accounting via the Unified Betting Account Center (BAC))	1 (specialized committee within a ministry)
United Kingdom	2 (GGR-based)	1 (moderate; “whistle-to-whistle” restrictions in sports broadcasting)	2 (national self-exclusion; broad responsible-gambling toolkit)	1–2 (site blocking and strong supervisory enforcement powers)	2 (independent gambling commission)
Spain	1–2 (GGR-based with segment variation)	2 (severe advertising restrictions since 2021)	1–2 (combination of statutory responsible-gambling tools and operator programs)	1–2 (systematic site blocking; evolving oversight of payments)	2 (specialized national gambling authority)
Italy	1–2 (relatively high GGR-based taxes and other levies)	2 (near-total advertising ban under the “Decreto Dignità”)	1–2 (national responsible-gambling measures and exclusion mechanisms, unevenly implemented)	1–2 (systematic site blocking; selective payment-related measures)	2 (specialized national gambling authority)

Ranges such as “1–2” indicate intermediate or mixed configurations between adjacent categories. Foreign entries reflect qualitative coding based on statutory texts, regulator reports, and secondary analyses cited in the article. The primary purpose of the table is to present the comparative framework and Kazakhstan’s relative position, rather than to quantify outcomes.

We organize regulation conceptually into five instruments—taxation, advertising controls, protection tools (exclusions), enforcement technology (website/payment blocking and centralized accounting), and regulator autonomy. These act through three main mechanisms—price and visibility, payment frictions, and monitoring/selection of operators—to influence two outcome classes: legal-market channelization and harm-related proxies (self-exclusions, debtor/public-official bans, recorded illegal-gambling cases). Expected directions, drawing on the public-health and gambling-policy literature, are as follows: competitive GGR-based taxation and strict but targeted advertising are expected to support channelization while limiting at-risk inflows; payment and website blocking should decrease recorded illegal-gambling cases; and centralized accounting should increase reported GGR and tax compliance.

Empirically, the analysis proceeds in three steps. First, we document pre−/post-reform changes in Kazakhstan’s indicators around the 2023–2024 reforms using the annual panel (Table 2) and a summary of 2024 levels and year-on-year changes (Table 3). Second, we use the coding rubric to position Kazakhstan relative to the selected European jurisdictions (Table 1). Third, we integrate these strands in the Discussion, using them to address the research questions in an exploratory manner and to derive evidence-informed, but explicitly noncausal, policy implications. Within this framework, the analytical expectations H1–H4 introduced in Section 1 function as heuristic guides rather than formal hypotheses. They indicate which combinations of instruments and outcomes we focus on in the descriptive analysis, but we do not test them statistically or claim to identify causal effects.

**Table 2:** Kazakhstan—annual values of key indicators, 2019–2024.

Year	Legal gambling services volume (bn KZT)	Gambling-specific tax receipts (bn KZT)	Criminal cases (illegal gambling, Art. 307)	Debtor-based exclusions (coverage)	Voluntary self-exclusions (coverage)
2019	13.6	n.a.	420	0	0
2020	89.7	n.a.	212	0	0
2021	501.2	11.9	225	0	0
2022	572.1	30.0	144	0	0
2023	368.7	15.9	101	0	0
2024	484.5	45.2	70	≈3,000,000	≈188,000

“n.a.” = data not available in a consistent published form at the time of writing. Legal gambling services volume is reported at current prices; values are converted from million KZT to billion KZT and rounded to one decimal place based on official national accounts. Gambling-specific tax receipts refer to the dedicated gambling-business tax; data are published from 2021 onwards. The 2019 value for criminal cases under Article 307 is derived from the official statement that 2024 registrations (70 cases) are six times fewer than in 2019; the 2023 value is back-calculated from the reported 30.7% year-on-year decline between 2023 and 2024. Zeros for debtor-based exclusions and voluntary self-exclusions indicate that the respective instruments were not yet in force or operational; both mechanisms were rolled out nationally only in 2024.

**Table 3:** Kazakhstan—key indicators in 2024 and year-on-year changes.

Indicator (outcome/proxy)	2024 value	Change vs. 2023	Interpretation
Legal gambling services volume	484.5 bn KZT	+31.4% y/y	Legal channelization ↑
Criminal cases (illegal gambling, Art. 307)	70	−30.7%	Enforcement impact / shadow ↓
Debtor-based exclusions (coverage)	≈3,000,000	+ ≈3,000,000 (new statutory exclusion; 2023 coverage ≈ 0)	Harm-reduction coverage ↑
Voluntary self-exclusions	≈188,000	+ ≈188,000 (new national register; 2023 coverage ≈ 0)	Harm-reduction coverage ↑
Offshore online market (est.)	4–5 × legal	n/a (no consistent 2023 baseline estimate available)	Displacement risk persists

Annual aggregates; sources: national statistics, regulator, and referenced reports. For debtor-based exclusions and voluntary self-exclusions, 2024 is the first year of operation of the respective instruments; 2023 coverage was effectively zero, so we report absolute increases rather than percentage changes. For the offshore online market, only approximate 2024 ratios relative to the legal segment are available; no harmonized 2023 baseline exists. All monetary values are in KZT (Kazakhstani tenge); “bn” denotes billions. For context, 1 USD≈457 KZT in 2023 (annual average exchange rate).

## 3 Results

Viewed over the longer period since 2009, legal gambling services rose from around KZT 3 billion to about KZT 571 billion in 2022, with two major growth spurts around 2010 and 2020–2021, followed by a contraction in 2023 and a partial rebound in 2024 that still remains below the 2022 peak. Over the same period, criminal cases under Article 307 declined from roughly 420 in 2019 to 70 in 2024 (−30.7% year-on-year), suggesting that the post-2023 developments form part of a longer trend of market expansion accompanied by gradual reductions in recorded illegal gambling.

Table 1 locates Kazakhstan relative to three large European jurisdictions on five regulatory instruments. Kazakhstan scores relatively high on advertising restrictiveness, protection coverage, and enforcement technology, but lower on the tax model (a heavy effective burden combining GGR, value-added tax (VAT), and fixed device levies) and regulator autonomy (a specialized committee within a ministry rather than an independent agency). This configuration shares features with Southern European models (such as Italy and Spain), which combine strict advertising bans and extensive responsible-gambling tools with comparatively high effective tax rates, rather than with the United Kingdom’s more liberal but strongly institutionalized system.

## 4 Discussion

Mechanism-by-mechanism comparison suggests that several elements of the European configurations are broadly transferable to Kazakhstan’s setting. Strict advertising limits and national exclusion systems map directly onto the 2024 amendments and registries; payment blocking and centralized accounting mirror Kazakhstan’s existing tools. The main differences lie in the tax mix and the degree of regulator autonomy. Given Kazakhstan’s recent trends (legal services up; recorded illegal-gambling cases down; protection coverage up), incremental gains might be achieved through gradual tax simplification toward more GGR-dominant bases and continued institutional strengthening, rather than wholesale import of any single foreign model.

Licensing is governments’ core instrument to control gambling: it defines permitted activities, eligible operators, and operating conditions. In Kazakhstan, the Law “On the Gambling Business” (No. 219-III, January 12, 2007) authorizes casinos, slot-machine halls, bookmakers, and totalizators; Article 6 prohibits all other formats, including online casinos, which therefore remain outside the licensing perimeter.

Kazakhstan’s model is geographically selective. Since 2007, casinos and slot halls may operate only in designated zones—the Shchuchinsk district (Akmola region) and the Kapshagay Reservoir shore in Konaev (Almaty region)—an approach also used in Russia since 2009 and intended to contain availability and associated harms. Establishments outside these zones are banned; by contrast, bookmaking and lottery activities may operate nationwide subject to licensing.

Internationally, gambling regulation has converged on licensing under strict conditions overseen by specialized authorities, with core objectives of excluding criminal involvement and protecting vulnerable consumers. Models range from state monopolies to multi-license systems; several Nordic jurisdictions, such as Finland, are now dismantling monopoly arrangements and introducing licensing for online gambling in order to curb migration to unregulated sites. Across these configurations, a central trade-off concerns whether tightly controlled online licensing can shrink the black market without increasing overall participation.

Gambling taxation is a key tool for raising revenue and managing industry growth. Legalization typically yields sizable fiscal returns, but, as discussed in more detail in Section 5.4, these receipts are usually regressive in incidence, raising normative questions about the appropriate degree of fiscal reliance on gambling-derived income.

Evidence supports a balance: rates should internalize social costs without pushing activity underground (Fiedler, 2015; Rockefeller Institute of Government, 2025; Tax Foundation, 2025). Several analyses point to an effective ceiling in the range of roughly 70–80% of GGR, above which legal operations become unsustainable and substitution to unregulated channels accelerates (Fiedler, 2015; Gambling Capital, 2020; Copenhagen Economics, 2016).

Kazakhstan applies a multilayered regime. First, a device-based tax sets fixed monthly rates under the Tax Code—for example, 1,660 MCI per gaming table, 60 MCI per slot machine, 300 MCI per betting counter/totalizator, and 3,000 MCI per electronic betting terminal—functioning de facto as capacity-based licensing fees. Here, MCI denotes the monthly calculation index, a standard unit in Kazakhstan’s tax code that is adjusted annually. Second, since 2023 a 20% GGR tax applies to stakes minus payouts. Third, operators withhold 10% personal income tax on gamblers’ winnings (often partly absorbed by operators). Fourth, gambling is subject to 12% VAT. Collectively, the effective burden approaches the approximate 80% GGR threshold.

The rationale is twofold: to capture revenue from a market exceeding 570 billion KZT in 2022 and to restrain excessive expansion. But heavy burdens risk shifting activity offshore or underground; optimal frameworks must keep legal offerings competitive with the shadow market (Philander, 2013; Taylor et al., 2024; Tax Foundation, 2025).

Comparative practice varies widely: in major regulated markets, effective GGR tax rates typically range from around 15–25% in jurisdictions such as the United Kingdom and Denmark to combined burdens approaching 40–50% in higher-tax environments. These differences reflect varying social tolerance of gambling and competition for investment.

Debate persists over instruments: percentage-based GGR taxes are viewed as fairer and more responsive, while fixed device rates are simpler and more predictable (Philander, 2013; Günay, 2023; Garrett, 2017).

Kazakhstan’s current tax configuration—high fixed device rates combined with a 20% GGR tax, VAT, and withholding on winnings—implies a very high effective burden that is likely to weaken legal channelization relative to jurisdictions with more moderate, predominantly GGR-based regimes (Table 3). These observations are consistent with the expected direction of association set out in H1, although the data remain descriptive and do not allow us to quantify the effect.

Gambling advertising is a sensitive policy area balancing industry interests and public protection. Most countries now accept that promotion must be tightly limited to shield vulnerable groups—especially youth—and to avoid encouraging excessive consumption. The World Health Organization recommends that gambling advertising and marketing be ended or substantially reduced (World Health Organization, 2023). Research shows that aggressive promotion by bookmakers, online casinos, and lotteries can normalize gambling and raise problem prevalence; hence advertising curbs are core “system-frame” measures alongside limits on accessibility and product design (Ukhova et al., 2024a).

Since the September 2024 amendments, Kazakhstan operates a near-ban on gambling promotion across television, radio, outdoor, and most online channels, with only narrowly defined exceptions (summarized in the Introduction). This places the country among the more restrictive jurisdictions internationally and broadly aligns its advertising regime with emerging public-health guidance.

Internationally, policy trends also point to stricter limits. In Italy, the 2018 “Decreto Dignità” introduced one of the strictest advertising regimes in Europe, effectively banning gambling advertising and sports sponsorship across media, including football shirt deals and most broadcast advertising (Italy, 2018; European Audiovisual Observatory, 2018; Ipsos UK and University of Bristol, 2024). Although the formal ban remains in place, ongoing policy debates in 2024–2025 consider a partial reopening of sports sponsorship subject to dedicated levies on betting revenue, and observers note that gambling brands still appear around football through indirect or foreign-facing advertising. Spain’s Royal Decree 958/2020 likewise imposes a highly restrictive framework, including tight time-of-day limits on broadcast advertising and a general prohibition of gambling sponsorship of professional teams, with some elements recently partially annulled by the Supreme Court but the core restrictions on football advertising remaining in force (Government of Spain, 2020; Aonso-Diego et al., 2025). In practice, both jurisdictions exemplify the tension between formally strict advertising bans and the persistence of cross-border or indirect marketing targeted at viewers during football matches. Viewers in both countries nevertheless continue to encounter gambling brands during football broadcasts and international streaming, which illustrates how nominally comprehensive bans coexist with persistent cross-border and indirect marketing.

Industry argues that moderate advertising by legal operators helps migrate consumers from illegal to regulated markets and that blanket bans can entrench incumbents and deter new entrants—as noted in Italy. Subsequent debates and industry submissions have called for a partial reopening of certain forms of sponsorship, arguing that strict bans may disadvantage domestic sport and channel advertising to foreign-facing platforms (Ipsos UK and University of Bristol, 2024). The World Health Organization also documents active industry resistance, including lobbying to influence policy and research (World Health Organization, 2023). The prevailing expert view treats gambling promotion unlike ordinary commercial advertising: a precautionary approach allows informational ads only in defined contexts, bans aggressive tactics, and prohibits targeting young or otherwise vulnerable audiences.

Evidence gaps persist regarding the precise effects of specific restrictions, but early indications suggest that reduced media visibility can lower recruitment from at-risk groups, especially youth. Because the strictest advertising bans only entered into force in the second half of 2024 and youth-focused prevalence data remain limited, H2 currently remains a forward-looking expectation grounded in international public-health evidence rather than something that can be evaluated empirically for Kazakhstan.

Social protection for gamblers is a core element of modern regulation at the intersection of public health and social policy. Vulnerable groups include people prone to gambling disorder, youth, and those for whom play entails heightened risks (for example, indebted individuals). International consensus holds that the state and operators share responsibility for harm minimization. Many jurisdictions codify responsible-gambling measures—age limits, self-exclusion, bet/time caps, and treatment programs (Wardle et al., 2024; Ukhova et al., 2024b; World Health Organization, 2023).

For analytical clarity, the instruments used to protect vulnerable groups can be grouped into three broad families: prohibitions, restrictions, and support. Prohibitions include age limits and categorical exclusions for high-risk categories such as indebted individuals or public officials. Restrictions structure how gambling may occur by imposing barriers at the point of access (registration and identity checks), constraining the intensity of play through precommitment tools that require players to set monetary and time limits in advance, and using automated behavioral monitoring to trigger warnings or temporary suspensions. Support measures comprise voluntary self-exclusion, information and education initiatives, counseling, and clinical treatment programs. Public-health frameworks increasingly advocate such multilayered architectures rather than reliance on any single instrument (Wardle et al., 2024; Ukhova et al., 2024b; World Health Organization, 2023).

Most countries set the minimum gambling age at 18 or 21. In Kazakhstan, casino entry and betting are limited to age 21, with 2023–2024 discussions about raising the threshold to 25 for casinos and bookmakers. In parallel, sales of energy drinks to those under 21 were banned, signaling a broader youth-protection agenda (Kursiv Media, 2024).

As outlined in the Introduction, recent reforms have introduced a national voluntary self-exclusion register covering all licensed operators. Early domestic uptake appears limited, but the legal basis is in place (Government of the Republic of Kazakhstan, 2024; Kursiv Media, 2024; The Astana Times, 2024). International evidence shows effectiveness when enforcement is strict; for example, the United Kingdom’s GAMSTOP system is widely used by problem gamblers (Hopfgartner et al., 2023).

The same reforms also introduced extensive statutory exclusion regimes for debtors and specified categories of public officials, military personnel, law-enforcement officers, judges, and managers of state-owned enterprises, primarily to prevent corruption risks and additional financial harm. This combination—excluding debtors (a highly vulnerable group) and conflict-of-interest categories—is rare globally and was justified as necessary for protecting citizens and raising industry responsibility (Kursiv Media, 2024). The approach aligns with targeted protections used elsewhere (for example, limits for social-welfare recipients).

In 2022, Kazakhstan adopted a clinical protocol for diagnosing and treating gambling disorder, yet only 15 cases have been officially recorded to date—likely reflecting underdetection and stigma. The World Health Organization calls for reducing stigma and ensuring accessible care (World Health Organization, 2024). Public-health research advocates integrated prevention (education, hotlines, therapy), often funded by industry levies—as in Australia, Canada, and Singapore (Ukhova et al., 2024b).

Protecting vulnerable groups therefore requires a portfolio of prohibitions, restrictions, and support measures rather than reliance on any single instrument. Kazakhstan’s 2024 reforms adopted many of these, combining strict bans for officials and debtors with self-limitation mechanisms. Debate persists over stringency: some warn that blanket exclusions may push play to illegal/offshore sites; others praise the proactive stance as socially responsible (EU Political Report, 2024; The Astana Times, 2024). The broader trend in best practice is clear—maximize protection from gambling harm while ensuring enforceable oversight.

In sociolegal terms, these exclusion and protection regimes are not neutral technical fixes but part of the power relations that define who may legitimately bear gambling-related risks. By banning certain categories (debtors, public officials) from legal gambling while maintaining a heavily taxed but licensed market for others, the state seeks to relegitimate the gambling economy as compatible with public morality and the integrity of public office.

Even the most sophisticated legislation fails if large-scale illegal gambling persists. Combating unlawful play is therefore a core public-policy priority worldwide. The illegal sector spans underground casinos and gaming halls, unlicensed online casinos and betting sites, and schemes that mask gambling as lotteries or “poker clubs.” As noted in the Introduction, online casino-style gambling is prohibited in Kazakhstan and therefore falls entirely into the “illegal” category, operating outside the national licensing framework. Academic and policy research highlights a “triple harm”: (1) exposure to fraud and unreliable payouts; (2) heightened social damage due to the absence of age checks and responsible-gambling controls; and (3) lost tax revenue that feeds the shadow economy (Wang and Antonopoulos, 2016; Banks, 2018; National Research Council, 1999; Williams et al., 2011).

The illegal online market has been sizable: despite a de jure ban, many international sites have continued to serve Kazakhstani users. Since 2019, the Cyber Surveillance system has blocked unlawful domains; by 2024, numerous brands (for example, Vavada, PinUp) were identified, blacklisted, and blocked at the regulator’s request. Yet blocking is imperfect because operators rotate domains, use VPNs, and deploy other evasions, so additional measures have been introduced.

Amendments in 2023–2024 strengthened criminal penalties. Operating online casinos became a distinct offense under revised Article 307, punishable by up to seven years’ imprisonment; sanctions also increased for underground casinos and unauthorized halls. A Comprehensive Plan 2024–2026 coordinates agencies (Government of the Republic of Kazakhstan, 2024). Banks and payment systems must block transfers to illegal-gambling accounts—an approach used in the United States and European Union to cut off deposits. In Kazakhstan these payment frictions are reinforced by the Unified Betting Account Center (BAC), which functions as a mandatory clearing hub for all licensed bookmakers: every online bet must be routed through the BAC, where it is verified, processed, and recorded before being settled with the operator.

In 2024, 70 cases were registered under Article 307—30.7% fewer year-on-year and around six times fewer than in 2019—trends that authorities link to tougher rules and systematic enforcement. At the same time, experts note that the underground market endures: illegal online turnover likely exceeds the legal sector by several multiples, with many wagers flowing to foreign sites and eroding the tax base.

Given the cross-border nature of online gambling, enforcement increasingly depends on international cooperation and shared technical standards, especially in relation to offshore platforms that can circumvent national website and payment blocking. Legal and criminological analyses emphasise that unilateral measures struggle to contain such markets and that cross-border jurisdictional conflicts are pervasive (Devaney, 2009; Jia et al., 2022; Ko, 2024). These challenges are further amplified by the rapid emergence of cryptocurrency-based gambling and hybrid payment models, which weaken traditional KYC/AML controls and complicate supervision (Gore, 2023; Torrance et al., 2023; Lansky, 2018; PwC, 2025). For Kazakhstan, deeper engagement in regional formats such as the Eurasian Economic Union, combined with participation in broader information-sharing initiatives and joint enforcement actions, is therefore likely to be more important than further tightening of purely national blocking measures.

Global practice suggests that legalization must be paired with determined enforcement; otherwise regulation remains largely symbolic (Chopin et al., 2024). Kazakhstan’s experience is broadly consistent with this logic: as blocking, prosecution, and financial oversight have intensified, recorded illegal-gambling cases have declined while the legal sector has expanded. At the same time, debates persist between advocates of strict prohibition and proponents of controlled legalization under close supervision, and the World Health Organization stresses that the appropriate balance between these approaches is context-dependent (World Health Organization, 2024).

These descriptive trends are broadly consistent with analytical expectation H3, which links the combined use of payment blocking and systematic website blacklisting to reductions in observed illegal activity. However, changes in enforcement priorities, prosecutorial discretion, reporting practices, and the migration of illegal gambling into harder-to-detect formats (including cryptocurrency-based platforms) may also contribute to the decline in recorded cases. In line with our overall approach, we therefore treat the alignment between H3 and the observed trend as suggestive rather than definitive and do not attribute the reduction in cases to the recent legal reforms alone.

Who should regulate gambling? Institutional arrangements differ widely by historical, legal, and cultural context, yet the literature highlights three main models: (1) a specialized state regulator (commission or agency); (2) division of functions among several ministries and agencies; and (3) frameworks that include nongovernmental or self-regulatory bodies under state oversight. Across jurisdictions with legal markets, a dedicated state authority is always present—a broad consensus reflecting the sector’s complexity and risks (Rorie, 2017; Cabot et al., 2017; Weidner, 2022).

In Kazakhstan, oversight initially sat within the Ministry of Culture and Sports. A 2023 reform created the Committee for the Regulation of Gambling and Lotteries within the Ministry of Tourism and Sports, consolidating licensing and supervision. The Committee now issues licenses, monitors compliance, sanctions violations, and coordinates action against the illegal sector. For online betting, Kazakhstan added a digital layer: the BAC, an authorized platform through which essentially all licensed bets must be routed. The BAC centralizes payment flows, retains a small fraction of each stake to cover processing and compliance costs, and maintains a real-time record of stakes and payouts for every operator. Technically, the BAC is integrated with national registers of voluntarily self-excluded individuals and debtors as well as with cybersurveillance and website-blocking infrastructure, so that transactions from listed persons or to blacklisted domains can be intercepted and blocked automatically.

In many mature gambling markets, regulation is entrusted to a specialized authority with statutory consumer-protection and enforcement mandates, such as the UK Gambling Commission; similar agencies operate in several European Union and Asian jurisdictions. Federal systems, including the United States and Germany, have historically distributed responsibilities across different levels and ministries, but recent reforms also move toward clearer allocation and consolidation of functions.

An independent or semiautonomous regulator can, in principle, better resist industry lobbying and apply rules impartially, but still depends on political backing. Funding arrangements based on industry fees may create perceived or actual conflicts of interest, as highlighted in debates around the UK model and the reform of Finland’s former monopoly system (Cabot et al., 2017; Sama and Hiilamo, 2024, 2025). Global practice increasingly favors a clear separation of roles: a regulator with strong consumer-protection and antiabuse mandates, and commercial operators obliged to comply.

The specialized Committee—though located within a ministry—already performs distinct regulatory functions, cooperates with law-enforcement bodies against illegal gambling, and drafts legislative improvements. This reflects international guidance, including from the World Health Organization (2023), that regulators should act not only as permitting bodies but as strategic centers for policy: monitoring services, enforcing rules, coordinating harm-reduction measures, and disseminating promising practices. Realizing this role in full will require adequate statutory powers, staffing, and analytical capacity.

International experience suggests broad consensus on core elements of gambling governance—licensing, taxation, advertising limits, player protection, enforcement, and a dedicated regulator—alongside persistent disagreements about implementation details (World Health Organization, 2023; Wardle et al., 2024). Kazakhstan broadly follows these trends while introducing distinctive features such as strict categorical exclusions for certain groups, a centralized betting-accounting system, and relatively high effective tax rates; these innovations warrant continued empirical evaluation for both effectiveness and unintended effects.

Centralized bet accounting via the BAC, which processes essentially all licensed betting transactions and is technically linked to exclusion registers and cybersurveillance tools, aligns with analytical expectation H4, according to which enhanced transaction monitoring should increase reported GGR, improve tax compliance, and sharpen the separation between legal and illegal payment flows. Testing this mechanism formally will require richer data and lies beyond the scope of the present study, but the institutional design is consistent with trends in jurisdictions that rely on real-time data for regulatory oversight.

## 5 Policy implications and recommendations

Building on the descriptive trends in Section 3 and the comparative analysis of regulatory instruments in Section 4, this section formulates a set of policy recommendations for Kazakhstan.

First, building on Section 4.1, we recommend completing the legal framework in a way that allows all major gambling formats, including currently prohibited online casino-style products and remote betting, to be brought under strict state supervision. In line with the experience of several European jurisdictions, a limited number of online licenses could be granted to operators that satisfy demanding capital, technical, and responsible-gambling requirements, provided that this step is accompanied by a predominantly GGR-based and internationally competitive tax mix and rigorous monitoring. Such a controlled opening would aim not at expanding gambling opportunities per se but at channeling a portion of existing offshore demand into the regulated sector, thereby strengthening consumer protection and fiscal transparency. Any licensing reform should proceed gradually and be subject to systematic evaluation of its effects on total participation, harm indicators, and the relative size of the illegal market.

Second, descriptive trends suggest that recorded illegal-gambling cases have declined, yet expert estimates indicate that offshore online turnover still substantially exceeds legal market volume. Rather than relaxing enforcement, this pattern points to the need for a more coherent and data-driven strategy against illegal operators. Priorities include maintaining and refining continuous monitoring of domains, strengthening cooperation with banks and payment systems on payment blocking, and implementing the 2024–2026 comprehensive plan in a way that emphasizes proportional but certain sanctions for organizers. Public communication campaigns should highlight the financial and legal risks associated with unlicensed sites to dampen demand. Given the scale and transnational character of offshore gambling, deepening participation in regional and international regulatory networks is therefore likely to be more important than further tightening of purely national blocking measures.

Third, the comparative coding in Table 1 underscores that Kazakhstan’s regulator has a narrower mandate and weaker autonomy than specialized gambling authorities in many European jurisdictions. We therefore recommend a gradual consolidation and strengthening of institutional arrangements rather than wholesale restructuring. Key steps could include clarifying the regulator’s statutory status and objectives, expanding its powers to conduct inspections and impose sanctions, and securing stable funding and technical expertise. Formal interagency coordination mechanisms that link the regulator with tax, law-enforcement, and health authorities would support more integrated monitoring of market developments and harm indicators. Participation in international regulatory forums can further enhance institutional learning and help benchmark Kazakhstan’s arrangements against evolving best practices.

Fourth, Kazakhstan’s multilayered combination of fixed device levies, GGR taxation, VAT, and withholding on winnings produces a high effective tax burden that may both weaken legal channelization and entrench regressive fiscal reliance on gambling revenues. A medium-term reform objective should therefore be to move toward a simpler, predominantly GGR-based tax structure with moderate rates aligned with those of comparable European jurisdictions, while containing the proliferation of overlapping charges. License fees and targeted contributions can continue to fund regulatory oversight and harm-reduction programs, but budgetary planning should avoid treating gambling receipts as a structurally indispensable revenue source. Evaluations of alternative tax designs should systematically consider their effects on illegal-market size, distributional incidence, and the long-term dependence of public finances on gambling-derived income.

Fifth, given the evidence on the role of promotion in recruiting new and at-risk gamblers, Kazakhstan’s precautionary approach to advertising should be maintained and refined rather than relaxed. Broad bans on mass-market advertising through television, outdoor media, and nonspecialized print appear justified on public-health grounds and should be complemented by stricter oversight of digital and social-media channels. Any permitted advertising ought to be confined to clearly age-restricted environments and accompanied by standardized health warnings in Kazakh and Russian. Sponsorship of sports and cultural events by gambling operators should either be prohibited or allowed only under tight conditions that limit the visibility of branding and require prominent harm messages. In parallel, responsible-information campaigns should present gambling risks and available support services in a clear, nonstigmatizing form.

Sixth, rapid expansion of exclusion tools in 2024 demonstrates Kazakhstan’s willingness to prioritize protection of vulnerable groups, but these outputs should not be equated with reduced harm. Policy development should focus on embedding exclusions within a broader portfolio of measures that includes rigorous age limits, a fully operational national self-exclusion system, standardized responsible-gambling tools (limits, reality checks, cooling-off periods), and accessible counseling and treatment services. In line with international recommendations, mandatory or strongly encouraged precommitment mechanisms for online and land-based gambling merit particular emphasis, as they can slow losses and prompt earlier reflection among regular gamblers. At the same time, categorical bans for debtors and public officials require ongoing evaluation for possible displacement effects. Culturally attuned public communication, including cooperation with trusted community and religious leaders, can help destigmatize help-seeking and reframe gambling disorder as a treatable condition rather than a purely moral failing.

Across these domains, the overarching principle is to maintain a dynamic balance between the channelization benefits of a regulated gambling market and the imperative to minimize gambling-related harm. Total prohibition risks entrenching illegal markets, whereas unchecked commercialization amplifies social costs and inequality. Kazakhstan’s recent reforms already move the system toward a public-health-first configuration; future adjustments to taxation, licensing boundaries, exclusion tools, and advertising controls should be guided by systematic monitoring of harm indicators and by transparent public debate about acceptable levels and distributions of risk and fiscal extraction.

## 6 Conclusion, limitations, and directions for future inquiry

This article has examined the evolution of Kazakhstan’s gambling regulation in comparative perspective, combining doctrinal and sociolegal analysis with a descriptive synthesis of official indicators for 2019–2024. Conceptually, we have treated gambling law not as a purely technical set of instruments but as a sociolegal regime that structures the allocation of gambling-related risks and fiscal rents between the state, licensed operators, and households. Drawing on risk-regulation scholarship, we have developed and applied an ideal-typical typology of liberal, restrictive, and prohibitive regulatory models, using it to locate Kazakhstan’s evolving configuration vis-à-vis selected European jurisdictions and to frame our empirical analysis.

Empirically, we have documented a pattern of rapid expansion of the legal gambling market, declining recorded illegal-gambling cases, and a sharp increase in the formal coverage of exclusion tools, alongside the persistence of a large offshore online sector. These descriptive trends suggest that Kazakhstan’s recent reforms have further channeled demand into the regulated segment and strengthened the institutional architecture of harm reduction, but also that substantial volumes of gambling activity remain outside direct state supervision. In addressing our research questions, we have interpreted these developments through the lens of regulatory instruments and their expected associations with market channelization and harm-related proxies, rather than as definitive estimates of changes in total social costs.

From a policy perspective, the emerging configuration combines strict advertising controls, extensive exclusion regimes, technologically intensive enforcement, and a specialized regulator with a comparatively heavy and complex tax mix and a regulator embedded within line ministries. This constellation is broadly aligned with public-health-first approaches increasingly visible in international practice, yet it also risks undermining the competitiveness of the legal market and entrenching fiscal dependence on regressive gambling-derived revenues. The central policy tension lies in reconciling the objectives of legal channelization, harm reduction, and revenue generation in ways that do not shift risks disproportionately onto lower-income households.

Taken together, our analysis supports a cautious, incremental reform strategy centered on public-health objectives: rationalizing gambling taxation toward simpler GGR-based structures, further strengthening regulator autonomy and technical capacity, deepening enforcement against offshore operators, and embedding exclusion tools within a broader system of prevention, treatment, and social support. Rather than offering a definitive blueprint, the study provides a sociolegal framework and a descriptive baseline to inform future empirical evaluations of Kazakhstan’s regulatory trajectory and its distributive consequences.

### 6.1 Limitations

This study has several limitations. Cross-country comparability is constrained by differences in legal definitions, reporting practices, and data availability. The post-reform observation window is short: only one full year of indicators (2024) is available after the major legislative changes, which precludes robust time-series evaluation and limits the scope for causal inference from pre−/post-reform contrasts. Illegal online activity is systematically undermeasured; proxies such as blocked sites and criminal cases capture only the most visible part of the market. In addition, reforms are themselves endogenous responses to underlying fiscal pressures and social concerns, so naïve associations between instruments and outcomes risk conflating causes and consequences. For these reasons, the analysis is explicitly descriptive: H1–H4 should be read as heuristic expectations that guide interpretation of descriptive patterns rather than as statistically tested hypotheses, and any apparent associations between regulatory instruments and outcomes cannot be interpreted as causal effects.

### 6.2 Directions for future inquiry

Future research can build on this descriptive baseline in at least four directions. First, extending the panel of indicators and applying quasi-experimental methods (such as difference-in-differences or synthetic control) would allow more rigorous estimation of the impacts of specific reforms, including advertising bans, payment blocking, and tax changes. Second, access to microlevel behavioral data from operators and self-exclusion systems would make it possible to analyze heterogeneity of effects by product type, risk profile, and sociodemographic group. Third, comparative work across post-Soviet and regional jurisdictions could clarify how different constellations of regulator autonomy, funding, and enforcement capacity mediate the relationship between regulatory instruments and harm outcomes. Fourth, the rapidly growing segment of cryptocurrency-based and decentralized gambling warrants systematic research, including on how such formats interact with existing licensing, blocking, and monitoring tools and what kinds of cross-border governance arrangements might be required.
